# Guided internet-based treatment for anxiety and depression in Norwegian primary care: a randomized non-inferiority effectiveness trial

**DOI:** 10.1016/j.invent.2026.100913

**Published:** 2026-01-25

**Authors:** Marit Knapstad, Otto Robert Smith

**Affiliations:** a Department of Health Promotion, Norwegian Institute of Public Health, Zander Kaaes gt. 7, 5015, Bergen, Norway; b Centre for Evaluation of Public Health Measures, Norwegian Institute of Public Health, PO Box 4404, Nydalen, N-0403, Oslo, Norway; c Department of Teacher Education, NLA University College, PO Box 74, Sandviken, 5812, Bergen, Norway

**Keywords:** Guided internet-based treatment, iCBT, Depression, Anxiety, Non-inferiority trial, Primary care, Acceptability

## Abstract

**Objective:**

Meta-analyses suggest that therapist-guided internet-based cognitive behavioural therapy (iCBT) is as effective as face-to-face CBT for anxiety and depression, yet its non-inferiority in routine primary care settings is scarcely examined. We examined the non-inferiority of the therapist-guided program “Assisted self-help” (AS-iCBT) compared to treatment as usual within the Norwegian Prompt Mental Health Care (TAU-PMHC).

**Methods:**

A pragmatic, parallel-group, randomized controlled non-inferiority trial with 1:2 (AS-iCBT: TAU-PMHC) allocation was conducted. Participants (*n* = 390, 37.4% of eligible) were adults considered for PMHC admission for anxiety and/or mild to moderate depression between October 2021, and December 2023. TAU-PMHC was predominantly individual face-to-face therapy (78%). Primary outcomes were change in symptoms of depression (PHQ-9) and anxiety (GAD-7) at six months follow-up. Symptom trajectories were analysed using latent growth curve models with robust maximum likelihood estimator (MLR), employing a non-inferiority margin of *d* > −0.30.

**Results:**

Both groups showed clinically significant reductions in PHQ-9 (*d*_w_: AS-iCBT = −1.15, TAU-PMHC = -1.26) and GAD-7 (d_w_: AS-iCBT = −0.92, TAU-PMHC = −1.11) at 6 months follow-up. However, AS-iCBT was not non-inferior to TAU-PMHC for primary outcomes (PHQ-9: *d*_b_ = −0.11 [95% CI -0.40; 0.19]; GAD-7: d_b_ = −0.19 [95% CI -0.43; 0.04]) and several secondary outcomes. Non-inferiority was demonstrated in client-rated but not therapist-rated alliance. AS-iCBT required approximately 46% less therapist time per client than TAU-PMHC.

**Conclusion:**

AS-iCBT did not meet the pre-defined criteria for non-inferiority compared to usual PMHC care for most outcomes. Nevertheless, AS-iCBT showed potential as a resource-efficient treatment option, requiring less therapist time while achieving clinically significant improvements. Further research should focus on optimizing its implementation, particularly for anxiety-related conditions.

## Highlights

• Guided iCBT was compared to usual primary care for anxiety and depression.
• Both treatments gave clinically significant improvements across outcomes.
• Guided iCBT was *not* non-inferior on primary and several secondary outcomes.
• Guided iCBT required approximately 46% less therapist time.
• Client-rated therapeutic alliance in guided iCBT was non-inferior to usual care.

## 1 Introduction

Adopting less therapist-intensive treatment modalities is considered a key strategy for reducing the treatment gap for anxiety and depression while maintaining a sustainable healthcare system (Thornicroft et al., 2017; Alonso et al., 2018). With internet being widely available, internet-delivered CBT (iCBT) is arguably among the most promising one. iCBT typically consists of a limited number of modules that the clients can complete by themselves. It can be delivered with or without therapeutic support, usually referred to as guided and unguided iCBT. The guidance can be delivered synchronous (e.g. via telephone) or asynchronous (e.g. via (messaging). A rapidly growing body of evidence supports the efficacy of iCBT for these conditions (Eilert et al., 2021; Olthuis et al., 2016; Harrer et al., 2025; Cuijpers et al., 2019), with guided iCBT showing particularly positive results (Harrer et al., 2025; Cuijpers et al., 2019). Though meta-analytic evidence even suggests equivalence between guided iCBT and other treatment modalities, including face-to-face CBT (Cuijpers et al., 2019; Hedman-Lagerlöf et al., 2023; Karyotaki et al., 2021; Carlbring et al., 2018), the bulk of studies have been designed as superiority trial, and not equivalence or non-inferiority trials, leaving it uncertain as to whether iCBT *is at least as* good as more traditional treatment approaches. Moreover, many studies comparing these treatment modalities have been relatively small or used open recruitment strategies. Clinical service recruitment trials (Romijn et al., 2019), including those in primary care (Wells et al., 2018), tend to show considerably weaker effects. As such, there is a need for more high-quality, large-scale trials of guided iCBT in routine primary care settings, including tests of non-inferiority compared to established forms of care (Harrer et al., 2025).

Within the British NHS Talking Therapies (formerly known as “Improving Access to Psychological Therapies (IAPT)) programme, perhaps the largest initiative worldwide to scale up low intensive treatment forms to improve access to care, two routine care superiority RCTs have been conducted on the effectiveness of guided iCBT. One compared iCBT programs for depression and anxiety with a waitlist control and found an effect in favour of iCBT on improvement in symptoms of depression (PHQ-9), anxiety (GAD), and functioning (WSAS) at 8 weeks of follow-up, with a further observed improvement at 12-month follow-up (Richards et al., 2020). The findings were also promising for cost-effectiveness, measured in quality-adjusted life years (QALYs) and resources used on healthcare services. The other trial compared guided iCBT for social anxiety with face-to-face therapy and waitlist (Clark et al., 2023). iCBT achieved outcomes comparable to face-to-face therapy post-treatment, and both outperformed waitlist. Moreover, iCBT required significantly less therapist-time, with only 6.45 h needed for iCBT to achieve symptom reduction comparable to 15.8 h in face-to-face therapy, or 2.45 times more symptom improvement per therapist hour (Clark et al., 2023). To date, no non-inferiority studies exist within “NHS Talking Therapies” on guided iCBT.

Widespread implementation of iCBT has been slower than expected, set against its promising results and potential. Existing evidence suggest that client satisfaction (Van Ballegooijen et al., 2014) and therapeutic alliance (Pihlaja et al., 2018; Sucala et al., 2012) in guided iCBT seem comparable to face to face CBT, with non-initiation in fact found lower in guided iCBT (Linardon et al., 2025). However, effectiveness studies often provide limited information on participant selection and the characteristics of those deemed unsuitable or declining guided internet-based treatment. A review of pre-post studies in routine clinical settings found moderate to high acceptability, measured by the characteristics of patients who initiated and completed treatment, and by patient satisfaction (Etzelmueller et al., 2020). There was, however, substantial variation in inclusion rates between studies and reasons for non-inclusion are frequently unclear (Etzelmueller et al., 2020). Moreover, the evidence on treatment completion are mixed, with study and treatment drop-out found be somewhat higher for guided iCBT in some (Cuijpers et al., 2019; Van Ballegooijen et al., 2014) but not all (Linardon et al., 2025) meta-analyses of RCTs. Better data on acceptability and a greater understanding of factors that can explain variation in acceptability are important for improving the implementation of internet-based treatment.

The current trial is conducted within the Norwegian version of NHS Talking Therapies, the primary care service Prompt Mental Health Care (PMHC). The trial was part of a pilot project initiated by the Norwegian Directorate of Health (NDH) to stimulate increased use of digital tools for treating anxiety and mild to moderate depression in primary care. The tool to be piloted, chosen through a tender competition, is developed and run by the company “Assistert selvhjelp AS” (English: Assisted self-help). Their tools are CBT-based and tailored to PMHC's target population and service. Although already implemented in most PMHC sites, uptake and implementation format varies substantially and have thus far only been evaluated in a qualitative study (Johnsen and Haddeland, 2021). The overall effect of PMHC has been tested in a trial with solid results, e.g. with a 6 months reliable recovery rate of 58.8% compared to 31.9% in treatment as usual condition (between-group effect size of 0.6) (Knapstad et al., 2020). Assisted self-help was not readily implemented in the sites by the time of the trial and the low-intensity treatment offer mostly comprised psycho-educational group courses.

The present paper extends findings from a report in Norwegian (Knapstad et al., 2025) with the primary objective to examine the non-inferiority of guided iCBT using “Assisted self-help” (AS-iCBT) compared to treatment as usual within the Prompt Mental Health Care service (TAU-PMHC), regarding effects on symptoms of anxiety and depression at six months follow-up. Secondary objectives: i) To assess the non-inferiority of AS-iCBT compared to TAU-PMHC on other outcomes such as quality of life, functioning, specific anxiety symptoms (social anxiety, panic disorder), sleep, and work participation; ii) Examine the cost-effectiveness (here: therapist time) of AS-iCBT compared to TAU-PMHC; iii) Assess whether AS-iCBT was as acceptable and appropriate (here: drop-out, therapeutic relationship, client satisfaction) of AS-iCBT compared to TAU-PMHC. Additionally, to further explore the acceptability and scalability of iCBT within PMHC, we compared background characteristics of individuals included and excluded from the trial and examined reasons for exclusion and patient treatment preferences.

## 2 Methods

### 2.1 Trial design and setting

The study was conducted as a pragmatic, parallel-group, randomized controlled non-inferiority trial with 1:2 (AS-iCBT: TAU-PMHC) allocation. The pragmatic design aimed for high generalizability to routine PMHC practice. The study setting was six PMHC sites covering 11 urban and rural municipalities in the east, west, mid and north of Norway (PMHC Lofoten [Vestvågøy, Flakstad, Moskenes], Fosen [Indre Fosen, Åfjord, Ørland], Karmøy, Sandnes, Notodden and Modum [Modum, Sigdal). The study was pre-registered on Clinicaltrials.gov (ID: NCT05118828, date of registration: 2021–10-17), approved by the Regional Committees for Medical and Health Research Ethics Southeast Norway (Ref: 254086) and is reported in alignment with the standard CONSORT guidelines with extensions for non-inferiority trials. Input on the design of the trial was provided by key stakeholders at all levels, from end-users (“Mental Health Association”) and service providers (all PMHC teams involved) to the national health authorities (NDH), and the trial was conducted in close collaboration with the participating municipalities.

### 2.2 Eligibility criteria, recruitment and randomization

Participants were eligible for inclusion if they were ≥ 18 years, resided in the service area of the pilot sites and presented with anxiety and/or mild to moderate depression, potentially co-occurring with sleep problems or early-stage substance use. These criteria align with the general target population of PMHC services, with the exception of the following exclusion criteria adjusted for the trial: (i) age 16–17 years; (ii) insufficient Norwegian language proficiency to complete questionnaires in Norwegian; (iii) inability or unwillingness to engage in iCBT; (iv) two or more previous treatment attempts in secondary care services for current complaints without effect; (v) severe physical health problems as primary concern; or (vi) previous treatment at PMHC and only required a booster session. Standard PMHC referral guidelines for more severe mental health conditions were also applied as exclusion criteria (e.g., severe depression, debilitating anxiety, risk of suicidal behaviour, eating disorders, bipolar disorder, psychotic symptoms, significant substance use disorders, and personality disorders). These criteria, except the one concerning ability to engage in iCBT, were similar to those applied in the trial that established the effectiveness of PMHC [20].

Participants were recruited among individuals seeking care at the PMHC sites. Intake assessment was conducted as per ordinary care by the PMHC therapists, by telephone or in person. PHQ-9 and GAD-7 were used to screen for mild to moderate depression and/or anxiety disorders, alongside a clinical interview. Cutoffs for PHQ-9 (≥10) and/or GAD-7 (≥8) served as guidelines, not mandates (no formal diagnosis provided). Eligible and consenting clients provided electronic informed consent and completed a baseline questionnaire. The baseline questionnaire included measures on socioeconomic background, work situation, health-related lifestyle and social support, treatment-related factors (such as reasons for help-seeking, treatment motivation, expectations and preferences), various process measures, and outcomes (see 2.7). Randomization was performed centrally by the researchers at NIPH after baseline data collection using a computer-generated random number sequence. Treating therapists were then informed of the allocation. Some site-specific adjustments in the allocation ratio were introduced due to recruitment challenges and practical requests from some sites, resulting in an overall ∼40%:60% split. This deviation from the original 1:2 ratio was documented in Clinicaltrials.gov.

### 2.3 Intervention and comparator

#### 2.3.1 AS-iCBT

Participants in AS-iCBT received treatment based on the Assisted Self-help program, consisting of six-part thematic CBT-based tools (https://assistertselvhjelp.no/verktoy-oppfolging/). Available tools addressed various common mental health concerns, with sub-modules incorporating psychoeducation, exercises, and assignments. Many tools share similar content but are presented differently for specific themes (e.g., ABC/cognitive therapy adapted for depression or anxiety). Participants were generally advised to complete one sub-module per week. Therapist were recommended to provide weekly support sessions. The sessions were delivered synchronous, preferably by phone or through video conference. While it is more common in the iCBT field to provide asynchronous support (Andersson, 2016), the content followed a typical set-up, by being short (should last 15–20 min) and focus on progress monitoring and feedback on completed work. On-site meetings were allowed as an exception based on clinical indications, but they should be minimized and focused on supporting program progress. One team deviated from this practice structurally as they wanted to have the first treatment session on-site (Fosen). During the pilot period, teams initially had access to six tools addressing depression, anxiety, social anxiety, panic disorder, sleep problems, and stress/strain. Some sites acquired additional tools (worries, self-esteem, return to work, perfectionism, health anxiety, phobias, and life management) during the data collection period. Since the study aimed to evaluate the format of guided internet-based treatment rather than the specific tools, participants at these sites could be offered these additional tools. In addition to the general PMCH training, therapist guidelines for each thematic tool were readily available in the AS system.

#### 2.3.2 Treatment as usual at Prompt Mental Health Care (TAU-PMHC)

The PMHC treatment model is adopted from the English NHS Talking Terapies and includes both low-intensity (guided self-help, psychoeducational courses) and high-intensity (short term face-to-face therapy) treatment forms, covering multiple CBT protocols for depression and a wide range of anxiety disorders. The care is delivered according to a matched care approach, in which the treatment offered is based on a decision reached jointly by therapist and patient. The duration of the treatment is tailored to the needs of the patient, with an upper limit of 15 sessions.

The TAU-PMHC group received standard PMHC treatment offered by their local service, excluding AS-iCBT to prevent contamination. The overall matched care model was applied across sites, while the specific organization and types of low-intensity treatment varied. For instance, some sites offered more group-based courses while one site delivered a different, paper-based self-help program in combination with on-site follow-up sessions. The specific treatments received by participants in this group are detailed in the results section.

### 2.4 Therapist training and attitudes

All therapists (*n* = 41) had minimum 3 years relevant university of university college education and each team included at least one clinical psychologist. They also had an additional 1-year basic training in PMHC model and cognitive-behavioural therapy, including specific training in AS-iCBT use. Before the evaluation commenced, most therapists (82.9%) had used AS-iCBT with some clients (mean 6–10). A pre-trial survey indicated on average slightly more negative than positive personal appeal of guided internet-based treatment (mean 2.8 [SD = 0.8] on a 5-point scale) and perceived suitability of this format for the PMHC target group (mean 2.8 [SD = 0.9]).

### 2.5 Outcomes

Unless otherwise specified, the outcomes were measured at each assessment (baseline, bi-weekly from 2 to 10 weeks, 6 months).

#### 2.5.1 Primary outcomes

The primary outcomes were change in mean levels of depression and anxiety scores from baseline to 6 months follow-up. *Depressive symptoms* were measured using the Patient Health Questionnaire-9 (PHQ-9), a 9-item scale with scores ranging from 0 to 27, higher scores indicate worse outcome. *Anxiety symptoms* were measured using the Generalized Anxiety Disorder-7 (GAD-7) scale, a 7-item scale with scores ranging from 0 to 21, higher scores indicate worse outcome. Both instruments have demonstrated high internal consistency (α > 0.80) (Knapstad et al., 2020; Kroenke et al., 2001; Spitzer et al., 2006).

#### 2.5.2 Secondary outcomes

*(Reliable) Recovery Rate:* Operationalised as scoring above clinical cut-offs (PHQ-9 ≥ 10 or GAD-7 ≥ 8) at baseline and below at 6 months, with reliable improvement (PHQ-9 change ≥ 6 or GAD-7 change ≥ 5) calculated based on the standard deviation and Cronbach's alpha of the baseline scores, following IAPT procedures (Clark et al., 2009). At baseline, 86.5% (*n* = 327) of the sample scored above clinical cut-off.

*Reliable deterioration:* In line with recommendations from Guidi et al. (Guidi et al., 2018), the percentages of clients by group who reliably deteriorated between baseline and 6-month follow-up were also reported, defined as an increase in PHQ-9 score ≥ 6 or GAD-7 score ≥ 5.

*General Functioning:* Assessed using the Work and Social Adjustment Scale (WSAS) (Mundt et al., 2002), a 5-item scale (0–40) measuring the impact of mental health problems across five domains, where higher scores indicate worse outcome.

*Health-Related Quality of Life:* Measured using the EuroQol 5-Dimension (EQ-5D) (Rabin and Charro, 2001) at baseline and 6-months follow-up. Scores from 5 to 25, lower scores indicate better outcome.

*Mental Well-being:* Assessed using the short version (7 items) of the Warwick-Edinburgh Mental Well-Being Scale (WEMWBS) (Tennant et al., 2007; Stewart-Brown et al., 2009) at baseline and 6-months follow-up. The short version has comparable psychometric properties to the long version (Stewart-Brown et al., 2009) and demonstrated high internal consistency in PMHC data (α = 0.83) (Smith et al., 2017). Scores from 7 to 35, lower scores indicate worse outcome.

*Social Anxiety:* The shortened Social Phobia Inventory (SPIN-9) as used (Johnson et al., 2017), measured at baseline and 6-months follow-up and analysed for those with clinically relevant baseline score (> 9, 63.9% (*n* = 241)). The internal consistency of the short version in PMHC data was high (α = 0.87) (Smith et al., 2016). Scores from 9 to 45, higher scores indicate worse outcome.

*Panic Anxiety:* Measured by the Panic Disorder Screener (PADIS) (Sunderland et al., 2017), measured at baseline and 6-months follow-up and analysed for those with baseline score ≥ 4 (40.7% (*n* = 154)) (Batterham et al., 2015). The scale assesses the frequency of panic symptoms over the past 30 days on a five-point scale and has demonstrated good validity and internal consistency (Batterham et al., 2015). Scores from 0 to 13, higher scores indicate worse outcome.

*Sleep Problems:* A simplified version of the Bergen Insomnia Scale (BIS) (Pallesen et al., 2008) was employed at baseline and 6-months follow-up, assessing difficulties with sleep onset, repeated awakenings, and daytime sleepiness. Participants were categorized as having insomnia based on DSM-5-like criteria.

*Physical Activity:* Three questions assessing frequency, intensity, and duration of physical activity, used in several large Norwegian population-based studies. Index (0–15) derived in line with the work from Kurtze et al. (Kurtze et al., 2008), where lower scores indicate less physical activity.

*Sedentary Behaviour:* Index (0–5) derived from to items assessing sedentary behaviour, based on the International Physical Activity Questionnaire - Short Form (Lee et al., 2011). Higher scores indicate more sedentary behaviour.

*Work Status:* Participants reported their current work status at baseline and 6-month follow-up using 14 categories. For analysis, these were dichotomized into (i) full- or part-time employment without sick leave and (ii) all other categories.

*Therapeutic Alliance:* Client-rated therapeutic alliance was measured using the Therapeutic Alliance Quality Scale (TAQS) (Bickman et al., 2010) bi-weekly from 2 to 10 weeks follow-up. TAQS is a 5-item scale (0–4) where higher scores indicate better alliance. The scale has satisfactory psychometric properties (Bickman et al., 2010).

*Treatment Satisfaction:* Participants rated their satisfaction with PMHC treatment on a scale from 0 (very little) to 4 (very large extent), measured bi-weekly from 2 to 10 weeks follow-up.

*Other Measures:* Therapist background, study exclusion reasons, therapy process (modality, format, duration, main problem, rating of the therapeutic relationship and clients' homework engagement), and treatment termination reasons (including dropout) were also recorded by the therapists. At baseline, participants were asked about their treatment preferences, motivation, and expectations for recovery.

### 2.6 Statistical analyses

Based on a non-inferiority margin of d = −0.30, 80% power, α = 0.05, and a 20% dropout rate, 129 participants in AS-iCBT and 258 in usual care (total *N* = 387) were required for the primary outcomes. To enable moderator and mediator analyses, a larger sample size was considered desirable.

Non-inferiority was concluded if the lower bound of the 95% CI for the standardized mean difference between groups at 6 months was d > −0.30. Latent growth curve models, with robust maximum likelihood estimator (MLR), were used to model symptom trajectories from baseline to the 6-month follow-up (7 time points) for the primary outcomes. Given the likely non-linear progression of symptoms, various model specifications were explored, including polynomial, piecewise, and free time parameter models, and combinations thereof. Model selection was based on chi-square difference testing and model fit indices (RMSEA <0.06 and CFI >0.95 considered acceptable, with changes in CFI ≥0.01 and/or RMSEA ≥0.015 indicating a significant improvement in fit (Chen, 2007)). Piecewise growth curve models with (partially) free time parameters were ultimately selected for both PHQ-9 (CFI = 0.96, RMSEA = 0.04) and GAD-7 (CFI = 0.98, RMSEA = 0.03).

A similar growth curve modelling approach was used for secondary outcomes with multiple time points, while linear slope models with variance constrained to zero were used for outcomes with two time points. Logistic regression was used to analyse the effect on dropout rates. Missing data for recovery rate were imputed using multiple imputations.

Intention-to-treat (ITT) and per-protocol (PP) analyses were conducted for primary outcomes. Given the pragmatic nature of PMHC without a fixed protocol, the PP analyses included participants who had at least 5 treatment sessions or recovered (based on client-reported PHQ-9 < 10 and GAD-7 < 8 up to 6-week follow-up or therapist-reported termination due to achieved therapeutic goals). Sensitivity analyses using two different missing not at random (MNAR) specifications (Diggle-Kenward selection model [MNAR1] and a pattern mixture model [MNAR2] (Enders, 2010)) were conducted for the primary outcomes. Fixed effects for municipality and treatment group were included in all models. For growth models, the intercept was not regressed on treatment group in line with Twisk's recommendations (Twisk et al., 2018) and the standardized between-group effect sizes (d_b_) are primarily reported with the pooled standard deviation at baseline as the numerator. A negative standardized between-group effect indicates that the estimated effect favoured the TAU-PMHC group, whereas a positive value indicates that the effect favoured the AS-iCBT group. For standardized within-group effects (i.e., changes over time), a negative value denotes a decrease in a given outcome score, and a positive value denotes an increase in that score.

## 3 Results

### 3.1 Participant flow and baseline characteristics

Between October 20, 2021, and December 31, 2023, 2201 individuals were assessed for eligibility. Of these, 75.9% (*n* = 1670) received PMHC follow-up, and 48.9% (*n* = 1077) met the study's inclusion criteria. Of the latter, 403 consented and were randomized (37.4% of eligible). Thirteen participants withdrew post-randomization, resulting in a net sample of 390 participants (155 in AS-iCBT and 235 in TAU-PMHC).

The mean age of the participants was 36.2 years (SD 11.6) and 69% were women. At baseline, the mean PHQ-9 score was 13.4 (95% CI 12.9–13.8), mean GAD-7 score 10.6 (95% CI 10.2–11.2), and 86.5% (95% CI 82.1–90.0) scored above the clinical cutoff on either the PHQ-9 or GAD-7 (Table 1).

**Table 1:** Baseline demographic and clinical characteristics.

	Overall (n = 390)	TAU-PMHC (*n* = 235)	AS-ICBT (*n* = 155)
Female sex	69.0 (269)	66.4 (156)	72.9 (113)
Age, mean (SD)	36.2 (11.6)	36.2 (11.2)	36.0 (12.2)
Higher education	42.5 (159)	43.2 (98)	40.4 (61)
Having a partner	75.5 (283)	76.0 (171)	74.7 (112)
Immigrant background	5.3 (20)	5.3 (12)	5.3 (8)
Work status			
Out of work	15.3 (58)	15.0 (34)	15.9 (24)
Employed, sick-listed	43.3 (164)	45.3 (103)	40.4 (61)
Employed, not sick-listed	41.2 (156)	39.6 (90)	43.7 (66)
Economic difficulties	17.9 (27)	20.3 (78)	22.5 (51)
Caseness	86.5 (327)	86.3 (196)	86.8 (131)
Indication insomnia	34.0 (128)	34.8 (79)	32.7 (49)
Antidepressants, daily	10.8 (31)	10.4 (18)	11.5 (13)
Sleep medication, weekly	9.8 (28)	8.6 (15)	11.6 (13)
Anxiolytic medication, weekly	5.7 (16)	5.9 (10)	5.3 (6)
Having elevated symptoms ≥6 months prior to baseline	77.8 (290)	78.7 (177)	76.4 (113)
Having symptoms at baseline level ≥ 6 months prior to baseline	42.3 (158)	43.8 (99)	39.9 (59)
Sought help for similar problems during the last 12 months prior to baseline	44.0 (165)	42.5 (96)	46.3 (69)
PHQ-9, mean (95% CI)	13.4 (12.9–13.9)	13.4 (12.8–14.0)	13.5 (12.7–14.2)
GAD-7, mean (95% CI)	10.6 (10.2–11.1)	10.6 (10.0–11.2)	10.6 (9.9–11.3)
WSAS, mean (95% CI)	19.7 (18.9–20.5)	20.0 (19.0–21.0)	19.3 (18.0–20.5)
WEMWBS, mean (95% CI)	13.3 (12.9–13.7)	13.5 (12.9–14.0)	13.0 (12.4–13.7)
EQ-5D, mean (95% CI)	5.5 (5.1–5.9)	5.6 (5.0–6.2)	5.4 (4.8–6.0)
SPIN, mean (95% CI)	12.5 (11.8–13.3)	12.4 (11.4–13.4)	12.7 (11.5–13.9)

Note: The descriptive statistics represent percentages (numbers) or means (standard deviations/95% confidence intervals). AS: Assisted selfhelp; EQ-5D: EuroQol 5-Dimension; GAD-7: Generalized Anxiety Disorder scale; PHQ-9: Patient Health Questionnaire; PMHC: Prompt Mental Health Care; SPIN: Social Phobia Inventory; TAU, treatment as usual; WSAS: Work and Social Adjustment Scale; WEMWBS: Warwick-Edinburgh Mental Well-Being Scale.

**Fig. 1:**
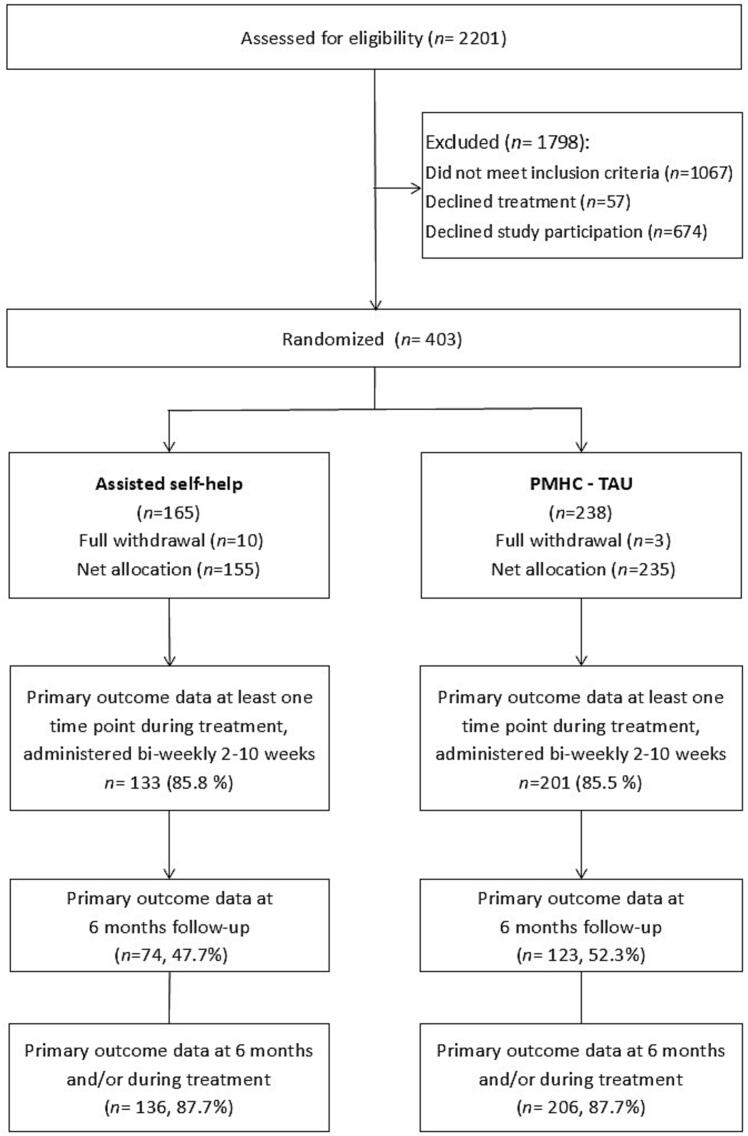
Flow diagram of participants. Inclusion period October 20, 2021, to December 31, 2023. AS: Assisted selfhelp; PMHC: Prompt Mental Health Care; TAU: treatment as usual.

Primary outcome data was available for 87.7% of participants in each group, defined as completion of the PHQ-9 and GAD-7 questionnaires at 6-month follow-up or at least one interim assessment. Approximately half of each group completed the 6-month follow-up questionnaire (See Fig. 1 for more details). Non-response rates at 6 months varied across sites and was lower among women, older participants (>35 years), and those with higher education. Baseline symptom scores were not associated with non-response.

### 3.2 Acceptability, scalability and treatment preferences

The 403 randomized participants constituted 37.4% of eligible and 24.1% of those receiving PMHC treatment during the trial period. Of the eligible non-participants, 53% (*n* = 362) declined because they did not want guided iCBT, while 7% (*n* = 47) specifically sought guided iCBT. The proportion declining iCBT notably varied substantially between sites (25%–67%). Of the non-eligible receiving PMHC, about half (55.8%, *n* = 319) were excluded for study-specific reasons (e.g. were enrolled for psychoeducational courses). The remaining were categorized as too mild (12.1%), too severe (12.5%) cases, or that guided iCBT was deemed inappropriate for other reasons (23.1%). Of the latter, the most common reasons provided were life crises, bereavement, trauma-related issues, cognitive challenges, and limited digital literacy.

There were only minor differences in general baseline characteristics between participants, eligible non-participants and the total PMHC client group, including sex and age-group composition, proportions having higher education, and in PHQ/GAD symptom levels (Table 2). The most notable difference was a lower proportion aged ≥60 among participants than non-participants.

**Table 2:** Demographic and clinical characteristics of participants and non-participants.

	Participants	Eligible non-participants	Non-eligible receiving PMHC	Non-participants receiving PMCH^†^	Declined iCBT*
	(*n* = 403)	(*n* = 674)	(*n* = 593)	(*n* = 1267)	(*n* = 362)
	%	%	%	%	%
Female sex	69.2	68.3	70.1	69.2	68.1
Age group					
≤30	34.0	34.0	31.4	32.8	31.5
30–60	63.5	56.9	58.9	57.9	58.8
≥60	2.5	9.1	9.7	9.4	9.7
Higher education	41.4	42.7	41.1	42.0	46.8
PHQ/GAD score					
PHQ and GAD <10	18.5	22.0	27.4	24.3	21.8
PHQ or GAD 10–14	36.3	31.9	28.5	30.5	31.0
PHQ and/or GAD >14	45.2	46.0	44.1	45.2	47.2

Note: ^†^Both eligible (n = 674) and non-eligible (n = 593). *Among eligible non-participants.

Among participants, nearly twice as many considered individual therapy (30.2%) to be the most suitable treatment form for them compared to guided internet-based treatment (17.0%), as measured pre-randomization. Over half (52.3%) reported that a combination of treatments would be best, had no clear preference, or were unsure. The majority (86.0% [95% CI 79.2–90.8]) were very or quite motivated to start treatment; two-thirds (67.1% [95% CI 59.0–74.4]) expected to recover and most participants on sick leave (82.6% [95% CI 75.3–87.9]) anticipated to return to work post-treatment.

### 3.3 Treatment characteristics and resource use

The median number of treatment sessions was 5 in both groups (IQR 3–7). However, the total therapist time per client was considerably lower in the AS-iCBT group (sample average 144.4 min) than in the TAU-PMHC group (sample average 266.0 min). The estimated average difference was 124.5 min per client (95% CI 97.8; 151.2). This corresponds to approximately 46% less therapist time per client in AS-iCBT than in TAU-PMHC. Therapeutic techniques, such as homework assignments, behavioural experiments, exposure training, and behavioural activation, were reported by the therapist to be significantly less frequently applied in the AS-iCBT group than in the TAU-PMHC group (*p* < 0.05 for all comparisons, Fig. A5).

*AS-iCBT:* In the AS-iCBT group 75% of sessions were conducted via telephone, with the remainder on-site (largely driven by Fosen having their first treatment session on-site as standard). Platform use was available for 58% of participants in the AS-iCBT group (*n* = 90). Among these, most (67%) primarily used one tool, with an average of 1.5 tools used (range: 1–4). The most frequently used tools were “Stress and strain” and “Depression,” both used by approximately one-third of participants, followed by “Anxiety” (21%), “Self-esteem” (17%), “Worries” (13%), and “Sleep problems” (6%). The median number of logins was 10 (interquartile range [IQR]: 6–16), with a median usage time of 3.2 h (IQR: 1.9–5.1) and completion of 42.5 treatment units (IQR: 27.5–54.5). A treatment unit is a subchapter within a treatment tool, with most tools having approximately 30 subchapters across six modules. Most participants appeared to have completed content equivalent to at least one full tool.

*TAU-PMHC*: In TAU-PMHC most treatment was individual therapy (78%), with relatively even distribution between group courses (12%) and guided self-help (10%). Most sessions in the TAU-PMHC group were conducted on-site (91.8%).

### 3.4 Primary outcome: changes in symptoms depression and anxiety

**Fig. 2:**
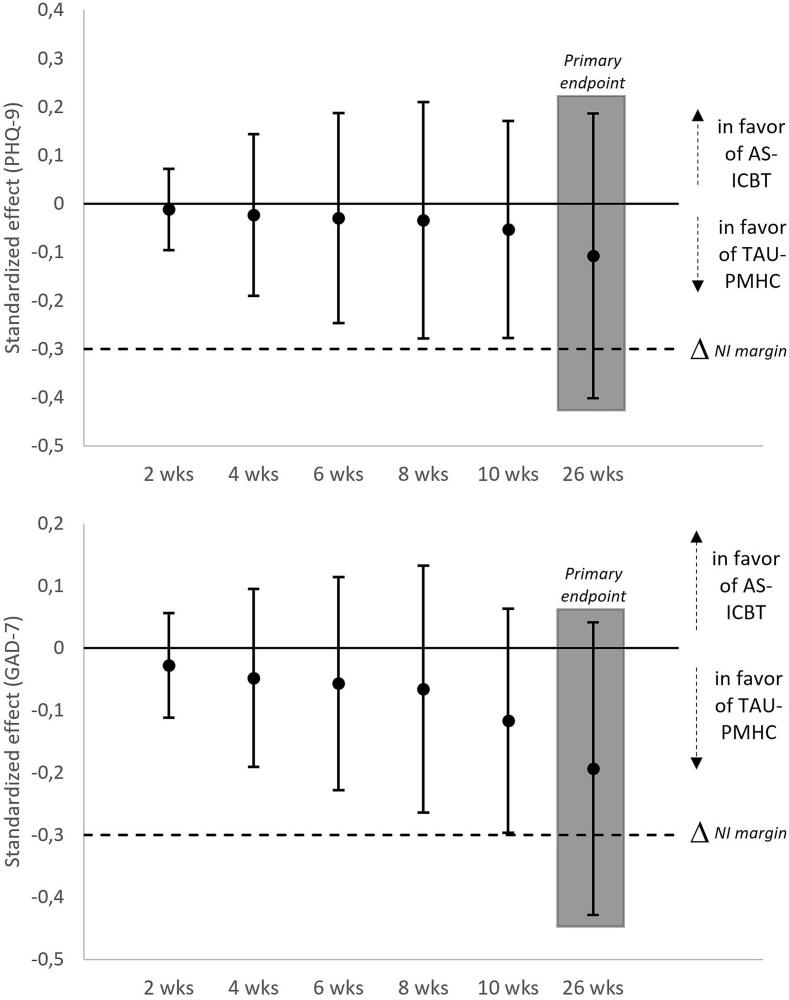
Standardized between-group effects over time for symptoms of depression (PHQ-9) and anxiety (GAD-7). Points estimates and 95% confidence intervals.

**Fig. A1:**
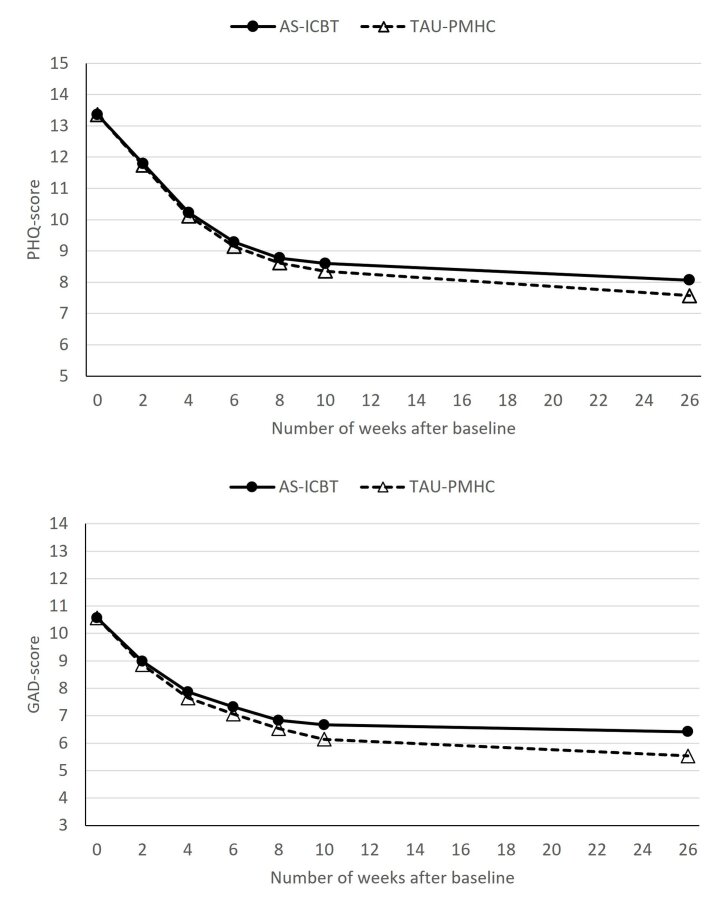
Change in PHQ-9 and GAD-7 score over time by treatment group. Intention to treat sample.

Both groups demonstrated clinically significant reductions in depression and anxiety symptoms at 6 months (Fig. A1). In the AS-iCBT group, the standardized within-group change (d_w_) was −1.15 (95% CI -1.40; −0.89) for PHQ-9 and -0.92 (95% CI -1.12; −0.71) for GAD-7. In the TAU-PMHC group, the change was −1.26 (95% CI -1.46; −1.05) for PHQ-9 and -1.11 (95% CI -1.29; −0.94) for GAD-7. The between-group effect size became more negative over time, favouring usual PMHC care. Non-inferiority of AS-iCBT compared to usual PMHC care was not established (> −0.30) for the primary outcomes at 6 months in the ITT analysis (PHQ-9: d_b_ = −0.11 [95% CI -0.40; 0.19]; GAD-7: d_b_ = −0.19 [95% CI -0.43; 0.04]) (Fig. 2).

**Fig. A2:**
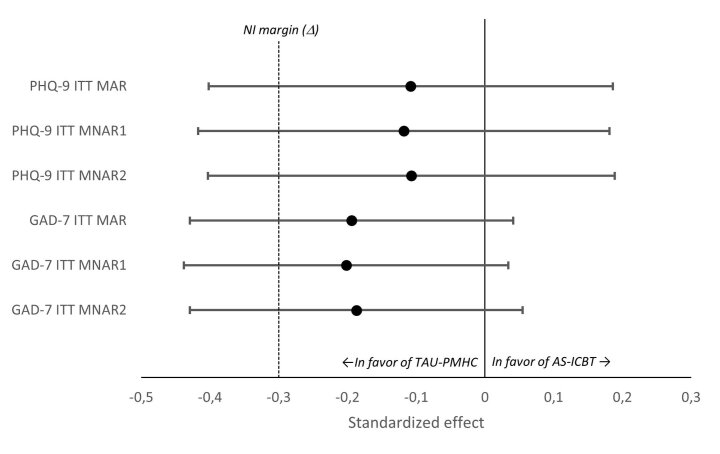
Between-group effect at six months follow-up based on various missing data assumptions for PHQ-9 and GAD-7. Points estimates and 95% confidence intervals.

**Fig. A3:**
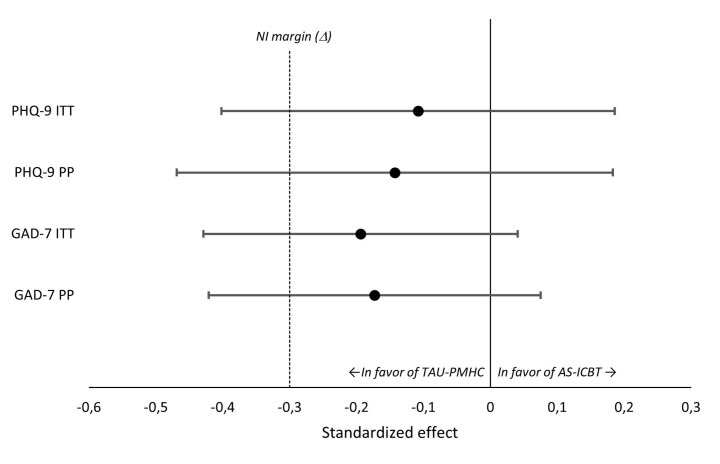
Between-group effect at six months follow-up based on the intention to treat (ITT) and the per protocol (PP) analyses for PHQ-9 and GAD-7. Points estimates and 95% confidence intervals.

Post-hoc sensitivity analyses using two different MNAR models yielded similar results to the main analysis, supporting the validity of the MI estimates under the MAR assumption. In the per-protocol (PP) analysis, 83.8% of participants in the TAU-PMHC group and 76.6% in the AS-iCBT group were included. The between-group effect sizes were even more in favour of the TAU-PMHC group (i.e. more negative), thus non-inferiority was not confirmed in the PP analysis either (Fig. A2, Fig. A3).

### 3.5 Secondary outcomes

Recovery rates were 56.1% in the AS-iCBT group and 58.9% in the TAU-PMHC group, and reliable recovery rates were 50.2% and 51.8%, respectively. Though the differences in point-estimates were minor, non-inferiority of AS-iCBT was not established (i.e., lower bound of the 95% CI for the standardized mean difference > −0.30). The estimated proportion of clients who reliably deteriorated was 1.3% (95% CI -2.6 to 5.2%) in the AS-iCBT group and 1.1% (95% CI -2.1 to 4.2%) in the TAU-PMHC group, which equalled a between-group effect size of −0.13 (95% CI –0.92 to 0.65, *p* = 0.74). No serious adverse events were reported. AS-iCBT showed non-inferiority to TAU-PMHC for mental wellbeing (sWEMWBS), social anxiety (SPIN), sleep problems and physical activity. Non-inferiority was not confirmed for general functioning (WSAS), health-related quality of life (EQ-5D), panic disorder (PADIS), sedentary behaviour and work participation (Table 3). The absolute within-group changes were greater than *d* = 0.75 for WSAS, sWEMWBS, SPIN and PADIS, whereas relatively limited in both groups, that is, lower than *d* = 0.5 in the other continuous outcome measures. Notably, TAU-PMHC was also not statistically significantly *better* than AS-iCBT for any of these measures.

**Table 3:** Between-group effects on secondary outcomes at 6 months follow-up (intention to treat-sample).

Outcome	AS-ICBT	TAU-PMHC	Between-group effect sizes
Recovery rate†, %	56.1 (45.1; 67.2)	58.9 (50.1; 67.6)	−0.06 (−0.38; 0.26)
Reliable recovery rate†, %	50.2 (39.3; 61.1)	51.8 (42.7; 60.9)	−0.04 (−0.36; 0.29)
WSAS, mean	13.9 (11.8; 16.0)	12.0 (10.4; 13.5)	−0.24 (−0.56; 0.08)
EQ5D, mean	4.5 (3.5; 5.4)	3.7 (2.9; 4.4)	−0.19 (−0.46; 0.09)
WEMWBS, mean	16.9 (16.0; 17.9)	16.4 (15.5; 17.4)	**0.12 (−0.19; 0.43)**
SPIN-9†, mean	10.5 (8.7; 12.2)	11.9 (10.5; 13.4)	**0.28 (−0.15; 0.71)**
PADIS†, mean	3.1 (2.0; 4.1)	2.7 (1.7; 3.6)	−0.18 (−0.81; 0.45)
Insomnia, %	24.3 (14.3; 35.7)	33.3 (24.6; 42.1)	**0.27 (−0.26; 0.80)**
Physical activity, mean	3.3 (2.8; 3.9)	3.3 (2.8; 3.7)	**0.02 (−0.18; 0.22)**
Sedentary behaviour, mean	1.8 (1.5; 2.1)	1.7 (1.5; 1.9)	−0.06 (−0.36; 0.24)
In regular work, %	64.4 (53.4; 75.3)	61.8 (53.7; 69.9)	0.10 (−0.34; 0.55)

Note: 95% confidence intervals in parentheses. Established non-inferiority in bold. †Only clients scoring above clinical cutoff baseline included in analysis.

**Fig. 3:**
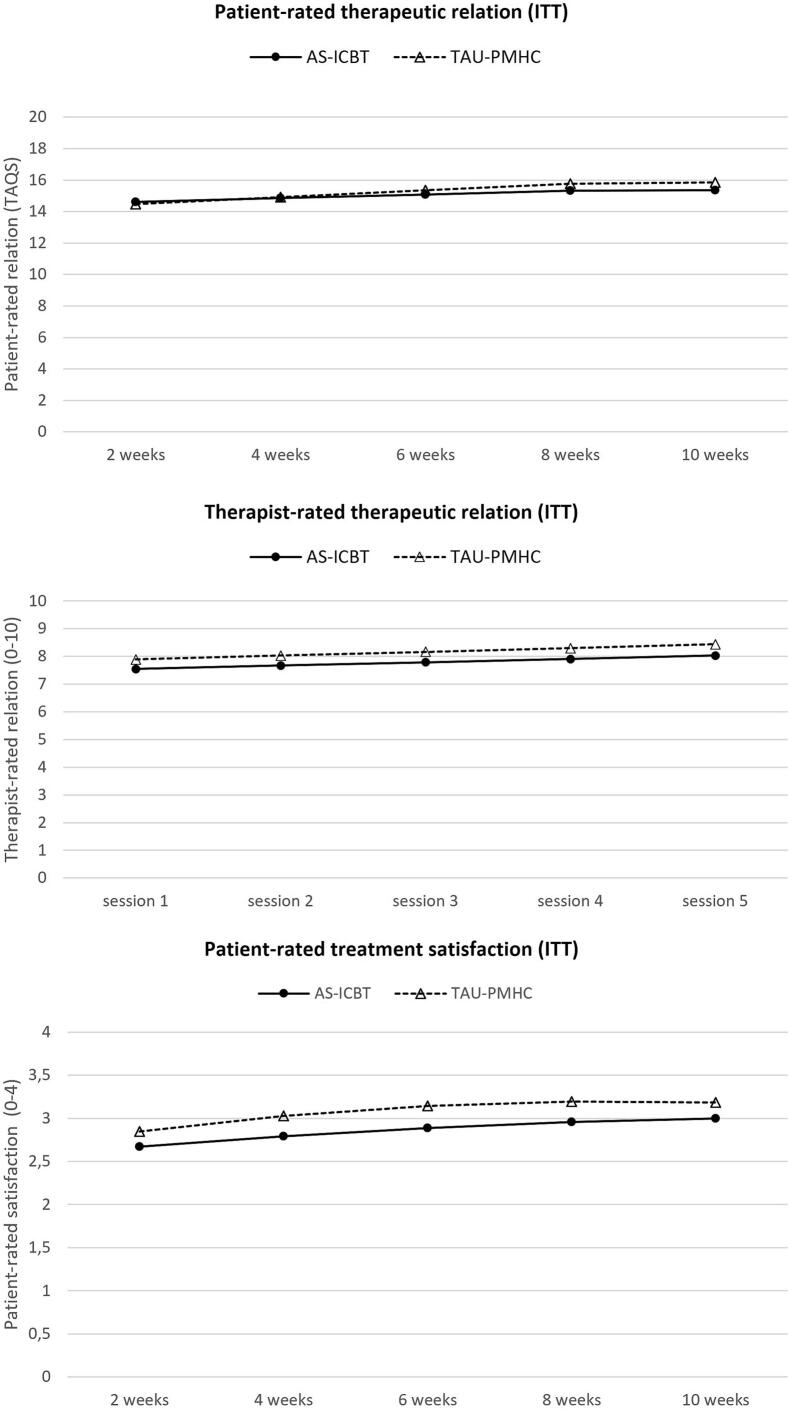
Rating of therapeutic relationship and satisfaction with treatment by over time by treatment group. Intention to treat sample.

Regarding therapeutic alliance, non-inferiority was found for client-rated, but not therapist-rated alliance (Table 4 and Fig. 3). Therapists contrarily rated the therapeutic alliance statistically significant better for clients followed up in the TAU-PMHC group those in AS-iCBT (Table 4). Clients receiving AS-iCBT were satisfied with the digital tools used. There was a tendency for slightly higher dropout rates among participants AS-iCBT group, and non-inferiority was not demonstrated. Early dropout (<3 treatment sessions) was 10.4% in the AS-iCBT group compared to 6.1% in the TAU-PMHC group (d_b_ = −0.31, 95%KI -0.73; 0.11). Drop-out based on per-protocol (<5 treatment sessions) was 23.5% in the AS-iCBT group versus 16.2% in TAU-PMHC (d_b_ = −0.25, 95%KI -0.54; 0.04).

**Table 4:** Between-group estimates for therapeutic relation and client satisfaction.

Outcome	Between-group effect size (95% C)
Client-rated therapeutic relation (TAQS, 0–20)	
2 weeks	**,04 (−,17;,26)**
4 weeks	**-,02 (−,21;,18)**
6 weeks	**-,08 (−,30;,14)**
8 weeks	-,14 (−,41;,14)
10 weeks	-,15 (−,45;,16)
Therapist-rated therapeutic relation (single item, 0–11)	
Session 1	-,26 (−,47; −,06)*
Session 2	-,28 (−,46; −,09)*
Session 3	-,29 (−,47; −,10)*
Session 4	-,30 (−,50; −,10)*
Session 5	-,31 (−,54; −,08)*
Client-rated satisfaction with treatment (single item, 0–4)	
2 weeks	-,20 (−,41;,02)
4 weeks	-,26 (−,45; −,07)*
6 weeks	-,28 (−,48; −,08)*
8 weeks	-,26 (−,48; −,05)*
10 weeks	-,21 (−,47;,06)

Note: Non-inferiority marked in bold. Superiority (of TAU-PMHC) marked with*.

### 3.6 Ancillary analyses

**Fig. A4:**
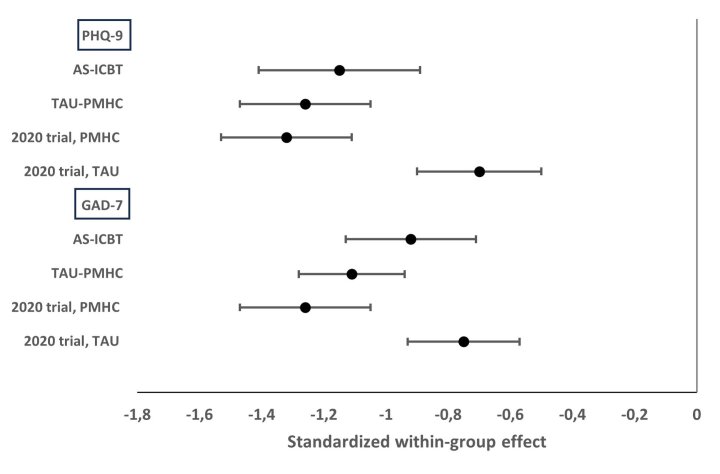
Standardized within-group effects for PHQ-9 and GAD-7 in the current trial and the previous trial of the PMHC, conducted in 2016–2017. Points estimates and 95% confidence intervals.

**Fig. A5:**
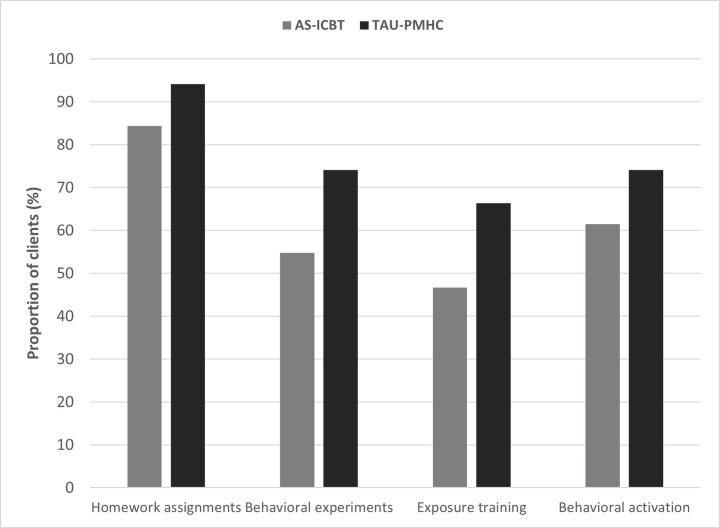
Therapeutic techniques by treatment group.

An exploratory analysis was conducted to compare the within-group effects of AS-iCBT six-month follow-up to the results of the previous trial of PPHC, where the control condition included regular primary care services such as GP consultations (Knapstad et al., 2020). Assuming similarity among the treatment groups, the AS-iCBT group would likely prove more effective than TAU at that point in time for depressive symptoms (PHQ-9), whereas effectiveness for anxiety symptoms (GAD-7) would be less certain (Fig. A4).

## 4 Discussion

Based on the predefined criteria, the current trial could not demonstrate that AS-iCBT was non-inferior to usual PMHC care for the primary outcomes of depression and anxiety symptoms at the 6-month follow-up. Similar results were found for most, but not all secondary outcomes. AS-iCBT was nonetheless associated with clinically significant improvements across outcome measures, most notably for depressive symptoms, with changes comparable to those in a previous trial of the PMHC service. The estimated (reliable) recovery rate for AS-iCBT exceeded 50%, the minimum standard for “NHS Talking Therapies” in England. Client-rated therapeutic alliance in the AS-iCBT group was furthermore at least as favourable as in the usual PMHC care group, while therapists rated the alliance as statistically significantly better in the usual PMHC care group. Finally, while both groups received relatively brief interventions (median 5 sessions), the AS-iCBT required approximately 46% less therapist time than usual PMHC care. Taken together, increased use of AS-iCBT in PMHC indeed could facilitate reaching more individuals with evidence-based treatment, given the relatively positive clinical outcomes.

Meta-analyses of RCTs suggest that guided iCBT for anxiety and depression is as effective as CBT delivered face-to-face (Hedman-Lagerlöf et al., 2023) and more effective than waitlist and usual care (Cuijpers et al., 2019). Very few have examined the non-inferiority (or equivalence) of guided iCBT in routine primary care settings, making direct comparisons difficult. Moreover, several of the studies included in meta-analyses have used group-based CBT as control condition, which may not yield results equivalent to individual therapy (Morrison, 2001). Further complicating comparisons with previous studies, the TAU condition in the current study included both individual and group treatments, though predominantly face-to-face treatment was delivered (78%). In the context of IAPT/NHS TT, the closest comparison is with within-group effect sizes from the trial by Richards et al. (Richards et al., 2020), which compared guided iCBT to a waitlist control. At 6-month follow-up, standardized within-group effects for the intervention group were approximately −1.34 for PHQ-9, and − 1.18 for GAD-7), aligning reasonably well with estimates in the current report (i.e., 1.15, and 0.92). Notably, the Richards et al. study only included participants scoring above the clinical cut-off on PHQ-9 or GAD-7 at baseline, which tends to inflate effect size estimates. Interestingly, similar platform use was reported in the current trial, with a median of 11 logins and 3.4 h tool user-time after eight weeks. While the treatment context was fairly similar, featuring online programs with 6 versus 7 modules and weekly support provided by similarly trained personnel, an additional difference was the provision of asynchronous support, so comparisons should be made with caution.

The estimated differences between AS-iCBT and usual PMHC care were slightly larger for anxiety (GAD-7) compared to depression (PHQ-9). Non-inferiority was notably demonstrated for social anxiety, but not for panic anxiety, and the decrease in GAD score from baseline to 6 months was somewhat less pronounced than in the previous trial of PMHC (Knapstad et al., 2020). Findings relating to anxiety were thus somewhat mixed and less certain. It should be noted that only a small proportion of participants (22%) had anxiety as their primary problem, and the heterogeneity of anxiety sub-types limited our ability to assess the effect of AS-iCBT on individual conditions. These results nonetheless partly contradicts general picture of larger treatment effects of digital interventions for anxiety conditions than depression (Harrer et al., 2025), and the limited literature suggesting similar effects of guided iCBT and face-to-face treatment for both social and panic anxiety (Hedman-Lagerlöf et al., 2023), although the evidence-base is insufficient to definitively establish non-inferiority (Harrer et al., 2025; Hedman-Lagerlöf et al., 2023). The therapist-reported lower use of CBT techniques in the AS-iCBT group compared to PMHC-TAU might have impacted the anxiety outcomes more than the depression outcomes. In line with this interpretation, some therapists found exposure training particularly challenging to implement in the AS-iCBT format (Knapstad et al., 2025).

The results regarding acceptability and scalability of iCBT within PMHC can be described as cautiously promising, with room for improvement. Few differences were observed between participants and non-participants in demographics and baseline symptom scores, suggesting some generalizability of the results to the broader PMHC target group. Compared to participants in previous trial of PMHC (Knapstad et al., 2020), where over 90% of eligible participated, the symptom profile was fairly similar, with only slightly lower symptom scores in the current sample and approximately the same proportion (88%) scoring above the cut-off for anxiety and/or depression. Nonetheless, participants represented only 24% of all PMHC clients during the study, with potential differences in other characteristics beyond these broad comparisons. Common reasons for exclusion included life crises, bereavement, trauma-related issues, cognitive challenges, and limited digital literacy. Therefore, caution is warranted in generalizing the findings to the entire PMHC population, though including a more diverse group of patients would not necessarily alter the results substantially (van der Lem et al., 2012). Based on reasons for exclusion it may be cautiously estimated that AS-iCBT could have been suitable for a considerably larger proportion than the 24.1% who participated, potentially at least 50%.

Unfamiliarity and scepticism toward guided internet-based treatment were evident, with 54% of eligible non-participants rejecting this treatment format and 17% of participants considering it their most suitable option. There was, nevertheless, substantial variation across sites in inclusion rates, and overall half of the participants favoured blended care, had no clear preference, or were unsure. This suggests that providing comprehensive information might help reassure and encourage patients to try this less familiar format (Vis et al., 2018). Despite initial, mixed expectations, treatment satisfaction was high in both treatment groups. Concerns that internet-based treatment formats might impede therapeutic relationships (MacLeod et al., 2009) were furthermore not supported, as both patient- and therapist-rated therapeutic alliances were satisfactory in both groups. The interesting discrepancy in therapist and patient ratings, where therapists but not patients rated the alliance higher when in the face-to-face format, was also found in the NHT TT study on iCBT for social anxiety (Clark et al., 2009). Like in the current study, the support sessions were conducted synchronous, mainly by phone. Finally, consistent with previous findings (Harrer et al., 2025; Cuijpers et al., 2019), there was a trend toward higher dropout rates in the AS-iCBT group. Like in a recent meta-analysis the differences in point-estimates were not statistically significant (Linardon et al., 2025), but non-inferiority was not demonstrated. Taken together, while the digital treatment format was fairly well received in the current trial, further research and efforts are clearly needed to enhance acceptance and adherence and to understand the therapeutic relationship's role when in guided self-help. Although the role of therapeutic alliance in adherence and outcomes in guided internet-based treatments is understudied, it seems crucial (Pihlaja et al., 2018; van Lotringen et al., 2021).

Engagement and mode of support are central yet complex issue in digital health interventions (Yardley et al., 2016; Cipriani et al., 2025). This study explored engagement using both objective (number of logins and hours of use) and subjective (satisfaction with the program) metrics. While these metrics are commonly used, their association with clinical outcomes remains uncertain (Yardley et al., 2016). More refined conceptualizations and models are being developed to better understand successful engagement, generally advocating for viewing engagement as a process and focus on what constitutes effective engagement, rather than merely its quantity (Yardley et al., 2016; Cipriani et al., 2025). An interesting avenue for research could involve exploring what constitutes effective engagement in interventions like AS-ICBT, for instance relating to the above-mentioned finding that therapists have found exposure training challenging. Understanding the relationship between effective engagement and outcomes could provide valuable insights. Additionally, the prevailing finding that guided iCBT tends to outperform unguided iCBT (Harrer et al., 2025; Cuijpers et al., 2019) indicates that human support plays a significant role in facilitating effective engagement. However, more detailed comparisons of the impact of various modes (such as synchronous vs. asynchronous) and the amount of support are less studied (Baumeister et al., 2014). Technological advances, such as automatic messages and chat-bots, introduce new complexities to these comparisons. The therapists reported positive experiences from initial in-person meeting with the patients (Knapstad et al., 2025), and as much as 25% of the support sessions in the AS-ICBT condition were conducted on-site, largely driven by one of the pilot sites systematically implementing on-site first treatment session. The impact of such meetings, as well as the difference between asynchronous and synchronous support, on alliance, adherence, engagement, and clinical outcomes should be systematically tested in an RCT (Vis et al., 2018). Relatedly, blended care (Erbe et al., 2017) might alleviate some of the potential drawbacks of guided internet-based treatment, such as lower adherence and higher dropout rates, while retaining advantages such as more efficient resource use. *Methodological considerations.*

Methodological strengths include the pragmatic study design, repeated measures, use of instruments with good psychometric properties. Conducting the study within routine PMHC services enhances the generalizability of the findings. Limitations include the failure to recruit the desired sample size in combination with higher attrition at 6 months-follow-up than anticipated, resulting in lower statistical power and limited possibilities for subgroup analyses. The non-response at 6-month follow-up increases risk of bias, although sensitivity analyses suggested robustness against alternative (MNAR) assumptions on missing data. The study commenced during the COVID-19 pandemic. This restricted face contact during the planning phase and capacity issues hindered the development of an optimal data collection system tailored to PMHC, potentially contributing to higher non-response.

The practical implementation of AS-iCBT may have influenced the results. Training in guided self-help and AS-iCBT was limited, with therapists encouraged to self-educate beyond the basic training, leading to variability in implementation. Some therapists reported feeling insecure and inadequately trained in AS-iCBT and expressed scepticism about its suitability for certain PMHC clients (Knapstad et al., 2025). The impact of these factors is uncertain, but implementation and attitudes may influence treatment outcomes (Romijn et al., 2019). Furthermore, the use of specific therapeutic techniques was reported to be less frequent in the AS-iCBT group, which may explain the smaller within-group effect on anxiety, as some techniques (e.g., exposure) are important for anxiety disorders. Therapist time was the only one factor considered in relation to cost-effectiveness. Questionnaire data will be linked with registry data healthcare use and benefit recipiency to conduct a more comprehensive cost-benefit and cost-utility analyses in a later paper.

## 5 Conclusion

This study did not demonstrate that AS-iCBT was non-inferior to usual PMHC care for primary outcomes. Nonetheless, AS-iCBT showed potential as a resource-efficient treatment option, requiring less therapist time while achieving comparable outcomes on several measures. Client satisfaction with AS-iCBT was generally positive. The findings support the use of AS-iCBT as part of the treatment options in services like PMHC to increase access to evidence-based treatment for anxiety and depression. Further research should examine how to optimize implementation, particularly for anxiety disorders, and include cost-effectiveness analyses.

## CRediT authorship contribution statement

MK and ORF contributed to the establishment of the research project to which the present study belongs. ORF and MK developed the design of the study. MK was PI and ORS responsible for coordination of the RCT. ORF carried out the main statistical analyses used in the present publication. MK drafted the manuscript, and both authors revised the manuscript for critical content and approved the final version.

## Ethics approval

The trial was approved by the Regional Ethics Committee for Western Norway (REK-vest, project number 254086) and pre-registered at Clinical Trials.gov (NCT05118828). All participants gave written informed consent.

## Declaration of Generative AI and AI-assisted technologies in the writing process

During the preparation of this work the authors used GPT University of Oslo to improve readability of the manuscript. After using this tool, the authors reviewed and edited the content as needed and take full responsibility for the content of the published article.

## Funding

The study received a grant from the 10.13039/501100014232Norwegian Directorate of Health. The funding body had no role in the design of the study and in writing the manuscript.

## Declaration of competing interest

The authors have no conflicts of interest to declare.
